# Community Health Representative Workforce: Meeting the Moment in American Indian Health Equity

**DOI:** 10.3389/fpubh.2021.667926

**Published:** 2021-07-21

**Authors:** Samantha Sabo, Louisa O'Meara, Kim Russell, Corey Hemstreet, J. T. Nashio, Brook Bender, Joyce Hamilton, Mae-Gilene Begay

**Affiliations:** ^1^Center for Health Equity Research, Northern Arizona University, Flagstaff, AZ, United States; ^2^Arizona Advisory Council on Indian Health Care, Phoenix, AZ, United States; ^3^White Mountain Apache Tribe Community Health Representative Program, Whiteriver, AZ, United States; ^4^Hualapai Tribe Community Health Representative Program, Peach Springs, AZ, United States; ^5^Hopi Tribe Community Health Representative Program, Hotevilla-Bacavi, AZ, United States; ^6^Navajo Nation Community Health Representative Program, Window Rock, AZ, United States

**Keywords:** community health representative, community health worker, American Indian/Alaska native, health systems, scope of practice

## Abstract

In 2018, the Community Health Representative (CHR) workforce celebrated their 50th year and serve as the oldest and only federally funded Community Health Worker (CHW) workforce in the United States. CHRs are a highly trained, well-established standardized workforce serving the medical and social needs of American Indian communities. Nationally, the CHR workforce consists of ~1,700 CHRs, representing 264 Tribes. Of the 22 Tribes of Arizona, 19 Tribes operate a CHR Program and employ ~250 CHRs, equivalent to ~30% of the total CHW workforce in the state. Since 2015, Tribal CHR Programs of Arizona have come together for annual CHR Policy Summits to dialogue and plan for the unique issues and opportunities facing CHR workforce sustainability and advancement. Overtime, the Policy Summits have resulted in the Arizona CHR Workforce Movement, which advocates for inclusion of CHRs in state and national level dialogue regarding workforce standardization, certification, training, supervision, and financing. This community case study describes the impetus, collaborative process, and selected results of a 2019–2020 multi-phase CHR workforce assessment. Specifically, we highlight CHR core roles and competencies, contributions to the social determinant of health and well-being and the level to which CHRs are integrated within systems and teams. We offer recommendations for strengthening the workforce, increasing awareness of CHR roles and competencies, integrating CHRs within teams and systems, and mechanism for sustainability.

## Introduction

**Figure 1:**
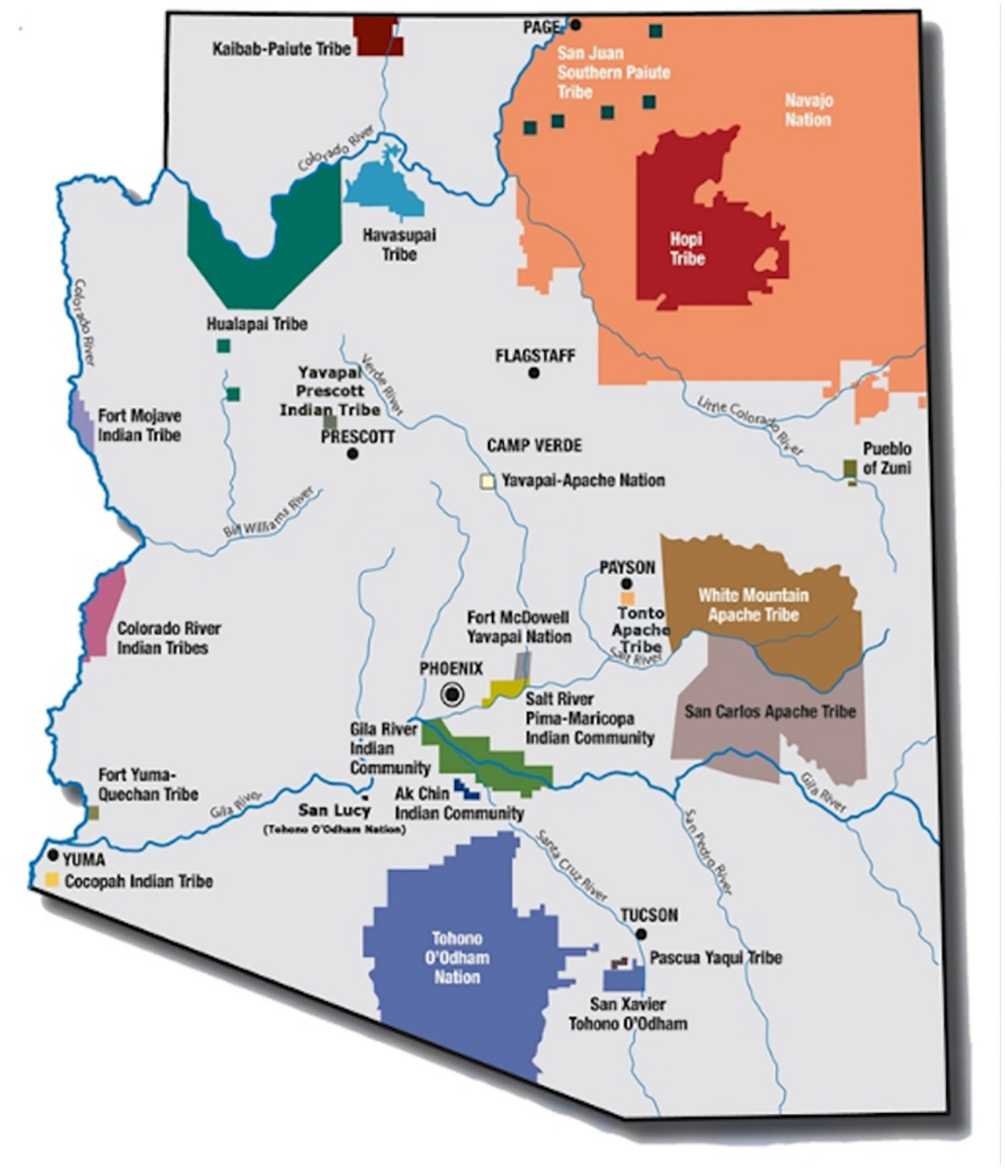
Native nations of Arizona.

In 1968, the Indian Health Service (IHS) funded the Community Health Representative (CHR) program through P.L. 100–713 as a component of healthcare services for American Indian and Alaskan Native (AI/AN) people (1). This policy established the first federally funded, community health worker (CHW) workforce, with origins in emerging anti-poverty and migrant health movements of the 1960s. In 1975, the Indian Self-Determination and Education Assistance Act, P.L. 93–638, facilitated Tribal authority to contract with the Federal government to operate programs and health systems serving their tribal members and other eligible AI/AN persons (2). Today, 95% of the 246 Tribal CHR programs (~2000 CHRs nationally) are tribally governed. In Arizona, the focus of this community case study, 19 of the 22 Tribes operate a CHR program, employing ~250 CHRs, equivalent to 30% of the total CHW workforce in Arizona [(3); Figure 1].

Since 2015, in direct response to statewide organizing efforts among the broader CHW workforce and allies, CHR programs of Arizona organized for annual CHR Policy Summits to dialogue and plan for the unique issues and opportunities facing CHR workforce sustainability and advancement (4–7). Over time, annual Summits resulted in an Arizona CHR Workforce Movement, which advocates for inclusion of CHRs in state and national level dialogue regarding workforce standardization, certification, training, supervision, and financing (8). Movement members include CHR Programs representing 19 Tribes, including CHR Program Directors, CHRs, health department directors, leading American Indian health and social policy entities, as well as state health department, Medicaid and university partners. Like many professional associations and conferences, annual CHR Summits and monthly CHR Movement meetings provide an interactive environment and mode of continuous communication among stakeholders in which policy initiatives and advocacy strategies unique to the CHR workforce can be discussed and deliberated.

Since 2015, the AACIHC has served as the backbone or convening agency for the Movement, with a larger mission to convene Tribal, state, and federal entities—including 22 representatives from each of the state's federally recognized American Indian Tribes—to advocate for increasing access to high quality healthcare programs for all AI/ANs in Arizona (6–8). CHR Movement members began to prioritize the need for CHR workforce assessments as an essential strategy to recruit, retain, and sustain a cadre of highly skilled, culturally and linguistically diverse CHRs. Moreover, CHR Movement leadership recognized the urgent need to better position the workforce in response to three important shifts in state and federal level workforce policy environments.

First and foremost, despite federal funding since 1968, CHR programs throughout the US are consistently called upon to demonstrate their effectiveness on health outcomes but have never in more than 50 years in operation had the resources to systematically collect the data necessary to demonstrate this level of impact. Such a challenge is in the light of overwhelming body of evidence of outcome and cost effectiveness of the broader CHW workforce across contexts and disease areas (9–12). Second, beginning in 2017 and culminating in fiscal year 2020, the Presidential Proposed Budget recommended phase out of the CHR Program and eliminating the health education programs funded in the IHS budget. Phase out was recommended in order to shift funds to extend the more medically focused Community Health Aide Program (CHAP) historically operating in villages of Alaska, to the lower 48 states (13). Third, after years of collaboration and collective advocacy, with critical advocacy efforts by CHR Programs and Tribes, Arizona CHW Voluntary Certification HB2324 legislation was signed into law on May 16, 2018 (14). Passing of this historic legislation, represented an essential opportunity to assure that the CHW/CHR workforce definitions were in alignment with all groups and that the scope of practice reflect CHW/CHR roles in both clinic and community-based settings (14). Thus, it was in this context that the first ever Arizona CHR workforce assessment was launched and serves to support current and future CHR professional development, training, supervision, career advancement, and financing of the CHR profession in Arizona (3).

Here we aim to highlight the collaborative process to engage the CHR workforce in identification of workforce development and sustainability priorities, and especially outline CHR core roles and competencies, contributions to social determinants of health and integration within systems and teams. Permissions have been obtained to reproduce some of the text published in our previous conference and assessment reports which are all located on the Arizona Advisory Council on Indian Health Care (AACIHC), website (https://bit.ly/306UscA).

## Context

Throughout the US, CHR Programs are organized and convened based on IHS designated Service Areas. For example, the 22 Tribes of Arizona are grouped into three distinct IHS Service Areas, including; Tucson, Phoenix, and Navajo Areas (15). Novel to our collective approach, the CHR Movement and CHR Policy Summits convene across the three IHS Service Areas. Beginning in 2018, in an effort to better understand the CHR workforce as a whole, members of the CHR Movement designed a preliminary CHR workforce assessment to be administered during an annual CHR Policy Summit. This conference-based assessment of the CHR workforce was the first of its kind in Arizona, and documented important demographic, professional, and training characteristics of the workforce across Tribal programs. This particular policy summit convened nearly 25% (*N* = 60) of the total CHR workforce employed in the state. Through this first step, we learned that among CHRs who attended the conference and completed the brief survey, that the CHR workforce in Arizona were predominately female, averaging 47 years in age with 13 years of employment experience as a CHR. Approximately one quarter of CHR survey respondents reported a high school diploma or a GED equivalent as their highest level of education, while almost half (47%) reported having achieved some college education and 23% had received a college degree. One quarter of CHRs reported an annual salary of less than $25,000 and ~53% of CHRs earned between $25,000 and $35,000 annually.

This conference assessment also illuminated CHR current and desired training. Standardized IHS CHR Basic and Advanced training requirements exist for the CHR workforce. Approximately 76% of CHRs reported having received Basic CHR Certification provided by the IHS CHR National Program. Approximately half of CHRs reported having had the opportunity to participant in IHS Advanced CHR trainings. Advanced CHR on line trainings include, motivational interviewing, case management, mental health, maternal, and child health and health promotion disease prevention modules. Approximately 63% of CHRs reported having completed an advanced CHR training in health promotion and disease prevention while 53% of CHRs reported completing modules in case management or mental health, with slightly less than half of CHRs receiving advanced training in maternal and child health. When asked if CHRs would like to receive advanced training in the future, 100% of CHRs wanted both Basic CHR Certification as well as all on line Advanced CHR Trainings offered by IHS. This conference evaluation had a profound effect on the partnership and sparked the collaborative workforce assessment efforts described in this community case study. Arizona CHR workforce assessments are robust in breadth and scope and reported in detail elsewhere (3, 7).

In the summer of 2019, as a result of the success of our conference-based assessment, the Arizona Advisory Council on Indian Health Care (AACIHC), at the guidance of the Arizona CHR Workforce Movement members, sought assistance from longtime university partners at the Northern Arizona University, Center for Health Equity Research (NAU-CHER) with experience conducting CHW workforce assessments, to conduct a multi-phase assessment of the CHR workforce in Arizona. The remaining case study highlights the process and results of Phase 1 and Phase 2 of the 2019–2020 CHR workforce assessment.

## Key Programmatic Elements

For purposes of this community case study, it is important to differentiate the CHR workforce and the CHR Program from the Community Health Aide (CHA) workforce and Community Health Aide Program (CHAP) (13). The CHA workforce consists of mid-level community, behavioral, and dental health paraprofessionals who provide healthcare services, including chronic, preventative and emergency care, to patients in tribal communities. The CHA program has been in place in Alaska since 1968 (16). In 2010 the Indian Health Care Improvement Act (IHCIA) was amended to authorize the creation of a national CHAP in order to expand the program to the lower 48 states (13, 17). This expansion remains in planning and development phase; however, in the last decade, a dozen states (including Arizona) have independently authorized the Dental Health Aide Therapist (DHAT) program component of the CHAP. IHS identifies three key areas that differentiate CHAs from CHRs: legislative authority, funding source, and scope of work. First, in regards to legislative authority, CHAP is authorized under 25 U.S. Code§ 1616l a-d, while the CHR program is authorized under the IHCIA public law 100–713 (13). Secondly, the two programs have different funding sources. While the CHAP in Alaska is funded through the IHS budget under the hospital and health clinics line item, CHRs are funded through a specific line item in the IHS budget. Finally, and most importantly, the scope of work for CHAs and CHRs are fundamentally distinct. Community Health Aides (CHA) and related Community Health Practitioners (CHP) are “mid-level medical providers” whose purpose is to provide basic medical care and connect patients with higher level medical care as needed (16). CHA/Ps function under the medical supervision of a licensed physician, through whom they are given authorization to treat patients, and follow a strict protocol to refer patients to higher medical care. The primary purpose of the CHR program on the other hand, is unique and distinct and in line with broader CHW workforce roles and competencies recognized by several federal entities, including: (1) Relationship and trust-building–to identify specific needs of clients, (2) Communication–especially continuity and clarity, between provider and patient; and traditional knowledge and language, and (3) Focus on Social Determinants of Health–conditions in which people are born, grow, work, live, and age, including social connectedness, traditional knowledge, and spirituality, relationship to the environment and a shared history.

Guided by the tenants of community based participatory evaluation (18) and using promising practices for assessing the CHW workforce (19) partners from NAU-CHER collaborated with the AACIHC and leadership of the Arizona CHR Coalition to define the scope of a multi-phase workforce assessment. In Phase I, CHR job descriptions and scopes of practice (SOP) documents were received from 12 of 19 Tribal CHR Programs, these documents were used to document current and emerging CHR core roles and competencies. In Phase II, collaborators developed a conversation guide for CHR managers to explore more deeply, CHR program organization, structure, financing, health system integration, and evaluation. In both phases all 19 CHR programs were invited to participate. Collaborators also intentionally or purposefully, identified, and recruited. CHR Programs that represented diverse programmatic characteristics, including service area settings, small and large population sizes, and public health and health care delivery program structures (i.e., contracted and compacted programs) to provide the greatest breadth of information for the assessment. NAU-CHER staff conducted 60-min telephone or video conference conversations with seven managers at six CHR programs. SOPs, job descriptions and conversations were analyzed for prominent themes using Atlas.ti Qualitative Analysis software. Table 1 outlines the specific goals and approaches to both phases of the 2019–2020 CHR Workforce Assessment. Although this assessment is not considered research, findings are confidential and responses are anonymous; information is reported in aggregate or as de-identified case studies to ensure anonymity of all participants and Tribes. In the following sections we describe major assessment topics.

**Table 1:** Arizona CHR workforce assessment objectives.

Phase I	1. Document current and emerging CHR core roles and competencies across the CHR workforce. 2. Establish a CHR workforce database to document workforce trends overtime (i.e., demographics, roles and competencies, career progression). 3. Compare CHR core roles and competencies across; (1) Tribal CHR Programs of Arizona, (2) Indian Health Service CHR Standards of Practice and (3) and National Community Health Worker Core Consensus Project.
Phase II	1. Document CHR Program organizational structure and financing. 2. Illuminate CHR core roles and competencies that address the social determinants of health. 3. Characterize the formal/informal relationships between the CHR Programs and Indian Health Service and 638 health systems and other Tribal health programs and sectors. 4. Assess current, planned and desired CHR Program process and outcomes evaluation.

### Characteristics, Qualifications, and Training

Phase I of the assessment documented several characteristics, qualifications, and training requirements (Table 2). Through an analysis of SOPs and job descriptions, CHRs were found to attain or possess various cultural, traditional, and linguistic experiences. All CHRs were required to have knowledge of the Tribe and community, including familiarity with the culture, traditions, health status, government, and socio-economic context. CHRs' required knowledge of the Tribe and community which is considered to translate to the CHR's ability to establish and maintain good working relationships with Tribal members, staff, IHS staff, and other Tribal departments and agencies. Approximately 58% of CHR Programs required or preferred CHRs to have the ability to communicate in the Tribe's language. Three quarters of CHR Programs required CHRs to be familiar with the local community and health resources available to clients. In accordance with Title VII of the Civil Rights Act, Sections 701(b) and 703(i) (20), 42% of programs identified a preference for CHR candidates who were of American Indian descent.

**Table 2:** CHR required and preferred competencies and skills.

**Required and Preferred Cultural and Traditional Knowledge and Skills**	
Knowledge of Culture and Tribe	100% (12/12)
Ability to Speak and Understand Language	58% (7/12)
Knowledge of Community Resources	75% (9/12)
Enrolled Tribal Member	42% (5/12)
**CHR Required or Preferred Formal Education and Training**	
CNA/CMA	75% (9/12)
Health Care Experience	92% (11/12)
FIRST AID/BLS	58% (7/12)
CPR	58% (7/12)
High School Diploma or GED	83% (10/12)
**CHR Training and Certification Provided Upon Hire**	
CHR Basic Certification^+^	58% (7/12)
RPMS/PCC	25% (3/12)
CAN/CMA	8% (1/12)
Fist Aide/CPR	17% (2/12)
HIPPA	17% (2/12)
Other Training or Certifications^*^	75% (9/12)

+ *Indicates in some cases requirement of the CHR Refresher course 36–48 months after completing the Basic CHR Training course*.

* *Evidence-based health promotion curricula or program*.

Three-quarters of CHR programs preferred a high school diploma or GED equivalent. Approximately 75% of programs required or preferred a Nurse Assistant (CNA) or Medical Assistant (CMA) certification and 58% of programs required or preferred a First Aid or Basic Life Support (BLS) and CPR certifications upon hire or within first year of hire. Approximately 92% of CHR programs required or preferred 6 months to 4 years of experience working in the health field, or in providing direct patient care or employment as a CHR. Most programs noted that any “equivalent combination of education and experience” that allowed the candidate to successfully perform the job duties would be considered. While, more than half (58%) of CHR Programs offered CHR Basic Certification upon hire through the IHS, only 25% of programs provided Patient Care Component (PCC) system coding and Resource and Patient Management System (RPMS) data entry training upon hire. No <75% of programs required or provided the opportunity for continued professional development through additional training or certification.

### Core Competencies and Scope of Practice

Phase I also focused on identifying the core roles and competencies of the CHR workforce in Arizona. To achieve this, we applied the National CHR standards of practice set by the IHS CHR Program and the national CHW Core Consensus Project (21) core roles and competencies to assess SOPs and job descriptions submitted by 12 participating Arizona CHR Programs (Table 3). The Indian Health Service published the Indian Health Manual, Part 3, Chapter 16 (22), which set forth the goals and objectives of the program, the standards of practice for the workforce, and requirements related to training, oversight, and data collection and reporting. IHS also published the RPMS Training Manual (23) which outlines the CHR service codes used by CHRs to document their services completed with individual patients, community organizations, and other events.

**Table 3:** CHR core roles and competencies by Indian Health Service and CHW Core Consensus Project.

**CHR Competencies and Roles (15)**		**CHW Competencies and Roles (24, 25)**	
Health Education	100% (12/12)	Cultural Mediation among Individuals, Communities, and Health and Social Service Systems	83% (20/12)
Case Find/Screen	100% (12/12)	Providing Culturally Appropriate Health Education and Information	100% (12/12)
Case Management/Coordinate	100% (12/12)	Care Coordination, Case Management, and System Navigation	100% (12/12)
Patient Care (Non-Emergency)	100% (12/12)	Providing Coaching and Social Support	83% (10/12)
Monitor Patient	100% (12/12)	Advocating for Individuals and Communities	83% (10/12)
Other Patient Centered Services	100% (12/12)	Building Individual and Community Capacity	42% 5/12
Transport	75% (9/12)	Providing Direct Service	100% (12/12)
Homemaker Service	75% (9/12)	Implementing Individual and Community Assessments	83% (10/12)
Interpret/Translate	67% (8/12)	Conducting Outreach	100% (12/12)
Environmental Health	50% (6/12)	Participating in Evaluation and Research	100% (12/12)
Emergency Patient Care	58% (7/12)		
Community Development	58% (7/12)		
**NEW CHR Roles**		**Fall Outside CHW SOP**	
Disaster Response	33% (4/12)	Homemaker services	75% (9/12)
Community Needs Assessment	33% (4/12)	Emergency Patient Care	58% (7/12)
Program Planning and Evaluation	67% (8/12)	Other Patient Centered Services	100% (12/12)

All 12 participating Arizona CHR Programs identified the CHR workforce core roles and competencies included the IHS standard of practice of: health education, case finding and screening, care management and coordination, and patientcare and monitoring. Approximately, 75% required homemaker and transportation roles, while 67% of CHR programs performed interpretation and translation roles. Approximately half of CHR Programs also identified environmental health, community development, and emergency patient care as CHR roles. All 12 (100%) CHR Programs identified the following national CHW core competencies of: (1) Providing culturally appropriate health education and information, (2) Conducting outreach, (3) Providing direct service, (4) Care coordination, case management and systems navigation, and (5) Participating in evaluation and research. One third of CHR SOPs included emerging roles, of community needs assessment and disaster response, and program planning and evaluation.

### CHR Integration Within Systems and Teams

**Figure 2:**
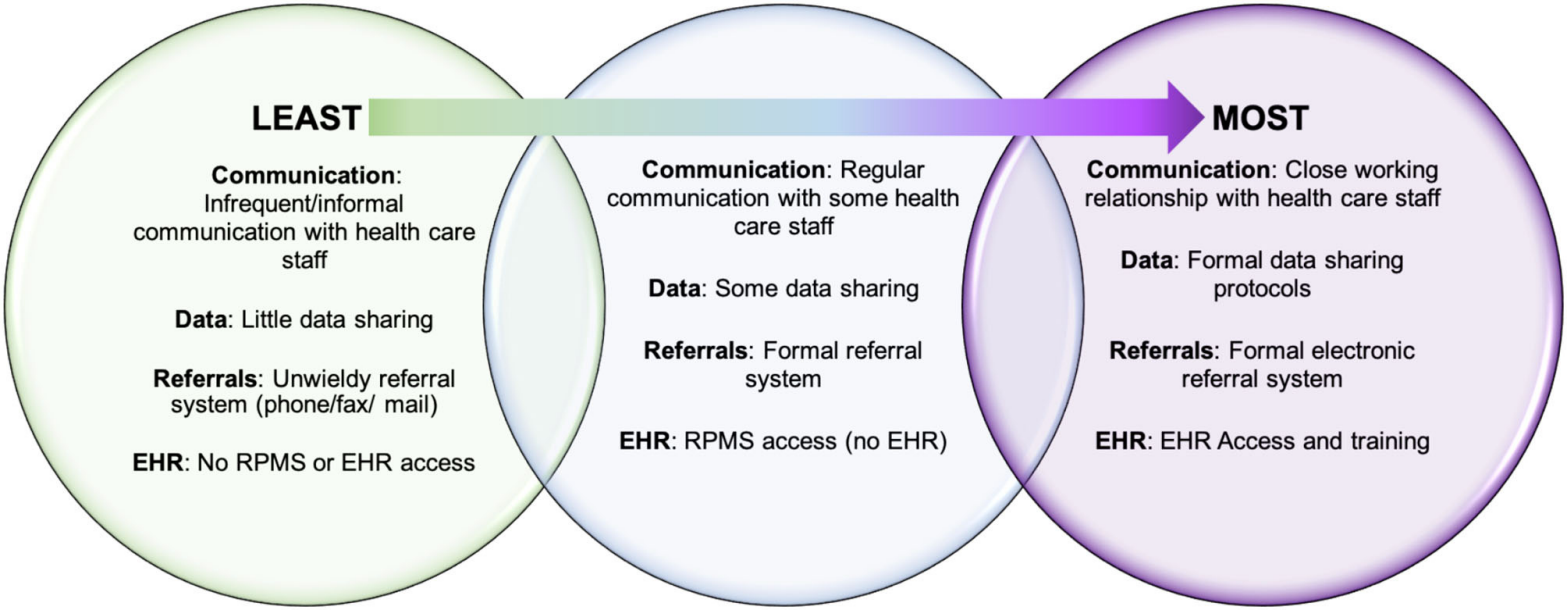
CHR-health system integration spectrum.

Phase II assessed CHR integration within systems and teams. According to conversations with CHR managers, the level of CHR integration with the IHS/638 health care systems varied among programs (Figure 2). In most cases, CHRs worked closely with public health nursing and met or communicated regularly with health facility staff to coordinate case management. Programs that had access to electronic health record (EHR), with the ability to enter notes and review patient charts, were afforded a higher level of integration. Conversely, programs without formal referral or data sharing systems in place were found less integrated into health care systems, resulting in CHR managers feeling that their programs were underutilized.

*The working relationship with the [IHS] service unit is integral in the delivery of health care services. Today, the CHRs play a critical role in the health care delivery system to link the patient to the IHS system and are intended to prevent avoidable hospital readmissions and emergency department visits through home visits to patients with chronic health conditions such as asthma, diabetes and hypertension*.

—CHR Program Manager

Complimentary to conversations with CHR managers, Phase I analysis revealed case management and care coordination as prominent CHR roles and competencies defined by three primary activities; service coordination, patient navigation, and advocacy. CHR competency and roles related to service coordination included coordinating patient/family centered services and home health services, with a variety of members of the health care team. CHRs were expected to work across providers and programs including primary care physicians, public health nurses, case managers, social workers, insurance case managers, dialysis clinics, local hospital, and other service providers. In some programs, CHRs were described to developed or execute patient care coordination and or discharge plans and were expected to be involved in chart reviews and monitoring of the patient. Some CHR were required to attend and participate in inter-agency care team meetings or staff meetings in which patient progress and plans were discussed and implemented by various members of that care team, including the CHR. For certain CHR Programs, service coordination required CHRs to coordinate and work closely with various federal, state, county, and local service agencies such as Arizona Medicaid, Arizona Long Term Care System, public health nursing, and Tribal programs. CHRs were designated to be both responsible for generating referrals, as well as receiving and following up on patient referrals. CHRs were expected to ensure communication between the health care team and patients, through delivering messages from the health care team and reviewing instructions for self-care. CHR collaborated with other departments, stakeholders and community groups to comprehend overall goals of the patient care plan, and planned outreach interventions and developed effective communication strategies between health care and social service entities and the patient and family.

Phase 1 also explored CHR care coordination, characterized as involving patient, community, and systems level advocacy. CHRs were expected to serve as the patient advocate through language translation and interpretation, arranging appointments, filing patient complaints, assisting the patient to obtain medication, medical equipment or transportation to ensure continuity of care. CHRs were expected to serve as an advocate for individuals and families by educating on available health programs, health policies and procedures; through assisting community members in seeking and applying for services through other resource agencies; and act as an advocate to communicate the needs of the clients to the medical team, CHR supervisor and public health nursing. CHRs roles also included advocating on behalf of both medical and social needs, such as light house cleaning and or cooking; completing necessary applications and or documents on behalf of the patient due to possible disabilities or physical limitations; picking up medications and delivering prescriptions and monitoring general health needs of the patient. Additionally, CHR roles includes acting as liaison and advocate for the community served by Federal, State and local agencies to improve the cultural responsivity and safety of the systems of care. This systems level advocacy included CHRs clarifying the role of American Indian traditional and cultural value systems, and cultural beliefs. Cultural and traditional advocacy supports the CHR Program goal to “reduce the potential for conflict and misunderstanding regarding the health conditions of American Indian and Alaska native people.”

Emerging within the role of care coordination was patient navigation. In some CHR Programs, SOPs and job descriptions articulated CHR ability to work with newly diagnosed clients, or clients with complex chronic conditions, including behavioral health diagnosis, substance use disorders or cancer. CHRs serving such clients were tasked with roles and competencies related to monitoring and support, including identification of the need for a higher level of care, emotional support for clients and their families with a chronic or serious illness or injury and referrals to the proper agencies for clients in crisis, clients experiencing loss, vulnerable clients, and other situations which affected family health and well-being. In some programs, CHR patient navigation activities also included helping clients identify a support network to provide for day-to-day care, arranging for transport of clients for follow-up care following discharge from a health, psychiatric, or residential substance abuse program, as well as transporting clients at high risk of deterioration in emotional or physical health.

### Challenges to CHR Integration

Despite robust descriptions of CHR roles and competencies related to integration within systems and team identified in Phase I, conversations with CHR program managers in Phase II, illuminated a number of challenges to integration of their programs within IHS/638 health systems. The two main barriers described by managers were a general lack of understanding about the CHR program on the part of health care staff, and a lack of communication and information sharing between CHR programs and providers.

#### Familiarity and Trust in CHR

CHR managers attributed the first issue of health care staff unfamiliarity with the CHR workforce in large part to the frequent turnover of IHS staff, often coming from off-reservation. One manager explained that this misunderstanding of CHR capabilities lead to an underutilization of valuable CHR services that extended healthcare into the community. In one case, where the CHR manager described ongoing issues related to health system integration, they pinpointed the heart of the problem as this misperception of CHRs among IHS staff:

*They don't really view them [CHR] as part of the system; they still view them as outsiders, more of a lay kind of employee with no technical skills, somebody that's a part of the community. And that's wrong – that's a misconception*.

The lack of understanding around CHR roles and responsibilities affected all aspects of CHR integration, from communication to case management. In addition, frequent staff turnover made it difficult to sustain relationships, particularly when the referral and communication processes are not formalized.

#### Communication and Information Sharing

The second area that CHR managers identified as presenting a significant barrier to health system integration was communication and information sharing, which included issues related to referrals, RPMS reporting, and belated involvement of CHRs in case management. Several program managers identified major gaps in the information-sharing process between CHRs and IHS/638 providers. Referrals were not always standardized and, in certain programs, were delivered via mail, fax or by hand, making them difficult to track systematically. Communication with providers was often by phone, on an as-needed basis, and because the RPMS is not connected to the EHR, the services that CHRs provide and health data they collect (such as blood pressure or blood sugar levels) are not seen by providers. In fact, providers may only be aware that their patient is receiving CHR services if the patient happens to mention it during a visit. One CHR manager, who is actively working to formalize their communication processes with IHS described the problem this way:

*[…] we serve the same patients that IHS serves. Why is it that we don't talk? Why is it that a CHR will do a journal entry into a patient's folder but yet, the doctor sees the same patient two days later and doesn't even realize that the CHR has taken screening vitals and that was good information for a doctor to look at? So, right now that's the challenge, is that our medical providers are not able to see the CHR notes. So, in a way I feel like our work is just being entered but who cares, nobody's going to use that data*.

This informal “as-needed” approach to communication also meant that CHRs were often contacted to assist with case management after a problem or crisis had emerged. As one CHR manager explained, CHRs were viewed as the “safety net” for patients, brought in to help resolve issues beyond the reach of standard health services, but not provided adequate resources or staff. One CHR manager explained how the health care system would benefit from greater CHR integration and involvement in primary and preventative care:

*So, they [the providers] will end up connecting with a CHR, but it always happens after the fact. […] if the CHR was actually integrated into the system, their response time would be much quicker, the patients would get quality care, they would get more out of communication with the provider. So, I think that's a misstep on health care systems*.

In spite of these barriers, CHR managers identified several strategies for improving CHR integration, described in the following section.

#### Opportunities for Improving CHR Integration

CHR managers discussed their efforts to address misunderstanding and misperceptions of CHRs among health system staff. To combat the volatility of frequent staff turnover, CHR managers actively worked to formalize communication and referral processes and advocated for their programs with health system leadership to improve CHR integration. One CHR manager who is relatively new to the position, described their efforts to bolster their program's sustainability:

*There's a lot of informal right now. Even on our referral process, so we really need to put that in black and white … I started advocating on behalf of the workforce, letting them know that we are an untapped resource yet we go into the community, we're boots on the ground, we are in the villages, week to week, and we know what's going on out there and we're able to assist*.

Another CHR manager suggested that CHR programs could use their position as the health care system's “safety net” as a point of leverage in building relationships with health system leadership and advocating for more resources. Several CHR managers pointed out that the responsibility for changing the current conditions should not fall exclusively to CHR programs. One CHR manager explained how IHS could proactively address the lack of knowledge about CHRs by requiring an orientation to Tribal programs for all new staff:

*I think there just needs to be some type of introduction to the Tribal programs. Especially the CHRs, so they can get a better idea and sense of how we're more of a resource for them, you know what I mean. I think that's something that needs to be changed and maybe integrated into IHS. I don't think they have a good understanding sometimes of what the CHRs are there for and how we can actually help them*.

CHR managers also frequently mentioned challenges related to the RPMS reporting system and expressed a desire for their CHRs to have access to the EHR. EHR access would allow CHRs to enter notes and vital information for providers to consider in patient care, provide a standardized trackable referral system, and improve CHR services by allowing them to review patient charts. One CHR manager described how EHR access has improved and facilitated the referral and information sharing processes with their 638-health care facility. They explained that while CHRs had been limited to basic data entry into the RPMS, with the EHR they are now able to more fully understand and contribute to their clients' care:

*And so now they have the capacity to read and understand what's going on with their patients, do some good chart reviews, that kind of thing about what's going on and we're actually starting to train them to put notes in. Because if they're doing the work, we shouldn't be getting in the way of them talking about what they saw and observed*.

## Discussion

CHRs are a highly trained, well-established standardized workforce serving the medical and social needs of American Indian communities. In Arizona, through a robust partnership across Tribal CHR Programs and key advocates in American Indian health policy, the CHR workforce remains coordinated and strong. CHR core roles and competencies make them a valuable member of the public health and healthcare system serving American Indian communities with the training, cultural, linguistic, and traditional knowledge to play a critical role in care coordination and case management. The degree to which each CHR program is integrated with the IHS/638 system is largely determined by the communication and information sharing practices in place. A significant barrier to full integration of CHRs into the health system is the common lack of understanding among IHS/638 staff of the roles of CHRs and the lack of formal protocols for communication and information sharing. CHR managers are actively involved in efforts to increase CHR-health system integration by educating partners about the CHR program, building relationships with IHS/638 leaders and advocating for greater CHR participation in teams. CHR managers identified two ways that IHS could improve CHR integration: first, to require an orientation for new staff to all Tribal programs; and second, to provide CHR programs with access to the EHR system to facilitate communication and care coordination between CHRs, providers and programs. Based on the workforce assessment results, the Arizona CHR Movement developed the following policy and environmental and systems recommendations to strengthen the CHR workforce in Arizona and nationally: (1) Increase awareness and acceptance of CHRs among the health care team by mandating orientation to CHR workforce competency, roles, and responsibilities for all medical and public health care staff; (2) Engage CHR Programs to establish a comprehensive evaluation system; (3) Establish procedures and policies for integrating CHRs as a functioning member of the health care team; (4) Establish a mechanism for reimbursement of CHR activities through state and federal Centers for Medicaid and Medicare; (5) Establish formal mechanisms for data collection and communication between CHR and public health and health care systems to ensure coordination of care and referrals among shared clients and patients; and (6) Support opportunities for CHRs to attain CHW voluntary certification through the state of Arizona. Nationally, the CHR workforce has earned the right to understand their collective workforce and its impact on the patient and population level health of the communities they serve. As a workforce, CHRs deserve to understand and plan for the financial, training, and workforce development of the next 50 years.

### Conceptual or Methodological Constraints

Experiences and case studies presented here do not necessarily present a complete picture of the range of CHR Program structures, activities, and health system relationships. Additionally, our analysis of existing CHR scopes of practice, job descriptions, and job announcements and conversations were limited to those CHR Programs of Arizona able to participate at the time of the assessment. Therefore, our analysis was restricted to what was outlined in the documents submitted by the CHR Programs, with some CHR Programs' documents more and less comprehensive, which may have resulted in under reporting of CHR roles and services, and or the lack of detail on roles and services unique to the CHR workforce. This assessment does not reflect CHR Programs in other IHS Service Areas or CHRs employed in non-IHS 638 Programs, such as Urban Indian Health Centers and or not-for-profit agencies serving American Indian populations. Despite these limitations, this workforce assessment is strengthened through its highly collaborative approach to data collection and interpretation of results by CHR Programs and American Indian health policy experts.

## Data Availability Statement

The datasets presented in this article are not readily available because original data is reported in publicly available reports only. Requests to access the datasets should be directed to https://nau.edu/cher/community-health-workers/.

## Author Contributions

SS and LO'M lead the writing of the community case study. KR and CH provided detail review and supported the development of public health policy recommendation. BB, JH, JN, and M-GB participated in the development of the workforce assessment protocol, and including its focus and conversation guides. Each contributed to the interpretation of original community health representative workforce assessment results and provided detail review of reports and reviewed the final versions of the community case study. All authors co-conceptualized the community case study based on the original community health representative workforce assessment results and reports.

## Conflict of Interest

The authors declare that the work was conducted in the absence of any commercial or financial relationships that could be construed as a potential conflict of interest. The handling Editor declared a past co-authorship with one of the author M-GB.
